# Cost-Effectiveness of Positron Emission Tomography/Computed Tomography (PET/CT) in the Initial N-Staging of Head–Neck Cancer and Comparison with CT and Magnetic Resonance Imaging (MRI)

**DOI:** 10.3390/curroncol32120677

**Published:** 2025-12-01

**Authors:** Nikolaos Papathanasiou, Maria Spiliotopoulou, Eleni Karagkouni, Dimitrios Apostolopoulos, Paraskevi Katsakiori, John Yfantopoulos, Nikolaos Kotsopoulos

**Affiliations:** 1 Nuclear Medicine Department, University Hospital of Patras, University of Patras, Rio, 26504 Patras, Greece; spiliotopouloumaria85@gmail.com (M.S.); dimap@upatras.gr (D.A.); 2 Radiology Department, University Hospital of Patras, University of Patras, Rio, 26504 Patras, Greece; lena726@gmail.com; 3 3DMI Research Group, Department of Medical Physics, School of Medicine, University of Patras, Rio, 26504 Patras, Greece; vkatsak@yahoo.com; 4 Department of Economics, National and Kapodistrian University of Athens, 15772 Athens, Greece; yfantopoulos@pspa.uoa.gr (J.Y.); nkotsop@econ.uoa.gr (N.K.)

**Keywords:** positron emission tomography (PET), PET/CT, head–neck cancer, cost-effectiveness, incremental cost-effectiveness ratio (ICER)

## Abstract

The aim of the study was to evaluate the cost-effectiveness of PET/CT in the initial N-staging of head–neck cancer (HNC) and to compare it with alternative strategies using CT or MRI within the Greek National Healthcare System. A cohort of 100 clinically N0 (with no apparent metastatic cervical lymph nodes) HNC patients was simulated over a 10-year time horizon. Initially, a decision tree model was used to simulate the following three different imaging strategies for HNC staging: (a) whole-body FDG-PET/CT, (b) CT of the neck, chest, and abdomen (“CT”), and (c) MRI of the neck plus CT of the chest–abdomen (“MRI”). Subsequently, a Markov model was used to simulate transitions into the health states of recurrence and death. Epidemiological evidence, diagnostic accuracy rates, transition probabilities, and healthcare costs were obtained from the literature and official local tariffs. The estimated total costs per patient were EUR 128,729 for PET/CT, EUR 128,779 for MRI, and EUR 128,585 for CT. The corresponding life years (LYs) were 6.171 LYs for PET/CT, 6.170 LYs for MRI, and 6.170 LYs for CT, respectively. The analysis showed that PET/CT dominates MRI. The incremental cost-effectiveness ratio (ICER) of PET/CT vs. CT was estimated at EUR 144,984 per LY gained. All three imaging strategies had comparable health outcomes and costs, with PET/CT being an appropriate and efficient imaging modality because of its high diagnostic accuracy in the N-staging of HNC.

## Simple Summary

This was a cost-effectiveness analysis of PET/CT for initial lymph node staging (N-staging) in head–neck cancer (HNC) within the Greek healthcare system. PET/CT was compared with CT and MRI over a 10-year period. Using decision analytic modeling on a cohort of 100 clinically N0 HNC patients, the analysis found almost similar clinical outcomes across all imaging approaches. PET/CT demonstrated costs of EUR 128,729 per patient with 6.171 life years (LYs) gained, compared to EUR 128,585 for CT (6.170 LYs) and EUR 128,779 for MRI (6.170 LYs). PET/CT marginally dominated MRI by providing slightly better outcomes at a lower cost, while showing an incremental cost-effectiveness ratio (ICER) of EUR 144,984 per LY gained vs. CT. Sensitivity analysis revealed that recurrence rates were the most influential factor affecting cost-effectiveness. Accounting for comparable costs, PET/CT’s high diagnostic accuracy makes it an efficient choice for initial HNC N-staging in resource-constrained systems, like Greece’s.

## 1. Introduction

Positron emission tomography/computed tomography (PET/CT) is a state-of-the-art, integrated, whole-body imaging modality that merges two complementary diagnostic approaches within a single examination, namely PET to trace metabolic activity at the cellular level combined with CT to provide detailed anatomical visualization [1]. Following its experimental debut in the 1990s, this fusion imaging modality has undergone significant advancements and has become integral to standard oncological care [2,3,4,5]. Currently, it is an essential component of routine clinical practice and directly influences therapeutic decision-making for oncologic patients. More than 90% of PET/CT studies use ^18^F-fluorodeoxyglucose (FDG) as the radioactive tracer. FDG is a glucose analogue, labeled with the radioactive positron emitter ^18^F, showing avid uptake by neoplastic cells. Cancer cells have high metabolic demands and primarily meet these needs via anaerobic glycolysis; hence, they demonstrate avid uptake of FDG. Once inside the cell, FDG is phosphorylated but not further metabolized, resulting in its entrapment within the cytoplasm and accumulation in malignant tissues [1,6,7].

FDG-PET/CT is universally endorsed by international clinical guidelines for initial head–neck cancer (HNC) staging, response assessment, and restaging [8]. Its superior sensitivity and specificity for nodal (N-staging) and distant metastases (M-staging) detection have been extensively documented [8,9,10,11,12,13]. PET/CT demonstrates superior diagnostic performance compared to CT and MRI in the evaluation of metastatic cervical lymph nodes, with sensitivity around 80–85% and slightly higher specificity [9,11,12]. The presence of metastatic nodal disease is a well-recognized adverse prognostic factor reducing survival by 40–50% [14]. Disease-involved nodes may show increased FDG uptake on PET and aggressive CT features, such as enlargement, rounded morphology, loss of fatty hilum, disruption of internal architecture, necrosis, clustering of three or more lymph nodes, and extracapsular nodal extension. The description of cervical lymph nodes follows a specific classification system into nodal levels, which ensures a common “map” among clinicians for optimal therapeutic patient management [15].

Advantages of PET/CT in N-staging include its superior ability to detect disease in small or normal-sized lymph nodes and nodes located in anatomically challenging regions, which are difficult to evaluate with clinical examination and conventional imaging modalities, such as the retropharyngeal space [9,12]. However, PET/CT has inherent limitations, like other imaging methods, to reliably detect micrometastases in occult lymph nodes due to spatial resolution constraints [16]. Cystic or necrotic lymph nodes may give false-negative findings, although this rarely occurs, because such nodes usually demonstrate increased metabolic activity at their peripheral margins. False-positive results occur in inflammatory or reactive lymph nodes exhibiting mild to moderate FDG uptake, which may mimic malignant involvement [9,11,12].

PET/CT is the modality of choice for the detection of distant metastatic disease and synchronous primary tumors [10,12,13]. Distant metastases are observed in ≥10–15% of HNC patients, most frequently in the lungs, skeleton, and liver. Locally advanced tumors (T3–T4), as well as those with a high nodal burden or extracapsular nodal extension, are associated with an increased risk of distant metastatic dissemination. In the post-treatment setting, oncological guidelines endorse PET/CT’s application for the accurate assessment of the neck following radical chemoradiation therapy [8,9]. Its diagnostic accuracy in the post-therapeutic setting is particularly valuable, since interpretation is often complicated by extensive surgical changes, including flap reconstructions, neck dissections, anatomical distortion, edema, asymmetries, and loss of normal fat planes. Furthermore, the high negative predictive value of PET/CT after radical chemoradiotherapy may spare patients from undergoing unnecessary and morbid nodal dissections [9].

Despite robust evidence supporting its diagnostic accuracy, the question arises as to whether the implementation of PET/CT is a worthwhile investment that provides the highest achievable value for the money spent. This question may be addressed through an economic evaluation, which assesses and compares alternative therapeutic strategies or diagnostic methods in healthcare [17,18]. For every euro allocated to PET/CT or other imaging modalities within the healthcare system, an inherent opportunity cost arises; specifically, these resources could have been invested in alternative interventions that may yield greater health benefits. The foregone benefit associated with not implementing those alternatives constitutes the opportunity cost. Such alternatives may include the funding of other imaging or diagnostic examinations, introducing novel treatments, implementing vaccination programs for infectious diseases, or investing in preventive health initiatives. Through economic analysis, proper resource allocation decisions systematically consider the comparative value and incremental health benefits among competing healthcare priorities. Specifically, within the Greek National Healthcare System, data on the cost-effectiveness of PET/CT are lacking. No prior economic evaluations have compared this approach with other conventional imaging modalities, such as CT and MRI, in this context. By means of decision analytic models, such as decision trees and Markov chains, we may gain valuable insights into resource optimization aimed at improving final clinical outcomes under the system’s current economic constraints [19,20,21].

This study conducted a cost-effectiveness analysis, tailored to the Greek healthcare setting, evaluating the use of PET/CT in initial HNC staging, with particular focus on cervical lymph node (N-staging). This modality was compared to alternative staging strategies including CT and MRI. The primary endpoint was overall survival in life years (LYs) achieved by different staging strategies: either with PET/CT or with CT-MRI and the cost per life year gained for the strategies under evaluation.

## 2. Materials and Methods

Economic evaluations compare the costs and benefits of healthcare interventions to assess the potential for economic efficiencies from introducing new therapeutic and/or diagnostic strategies. Gains are often expressed in terms of quality-adjusted life years (QALYs); however, in this analysis, due to the lack of quality-of-life data, using life years (LYs) gained was deemed appropriate. LYs are a metric of survival benefit which is an important metric in oncology. In health economic evaluations, particularly cost-effectiveness analyses, such as the present study, the assessment of economic efficiency is conducted by calculating the incremental cost-effectiveness ratio (ICER) [22]. This metric provides a quantitative framework for comparing a new intervention with the existing standard of care by relating the difference in costs to the difference in clinical outcomes, which are expressed as LYs gained or QALYs. The ICER is calculated using the following formula:

$$ICER=\frac{C_{2}-C_{1}}{E_{2}-E_{1}}=\frac{\Delta C}{\Delta Ε}$$

in which C_2_ denotes the cost of the new treatment, C_1_ is the cost of the standard treatment, Ε_2_ is the outcome of the new treatment and Ε_1_ is the outcome of the standard treatment.

**Figure 1:**
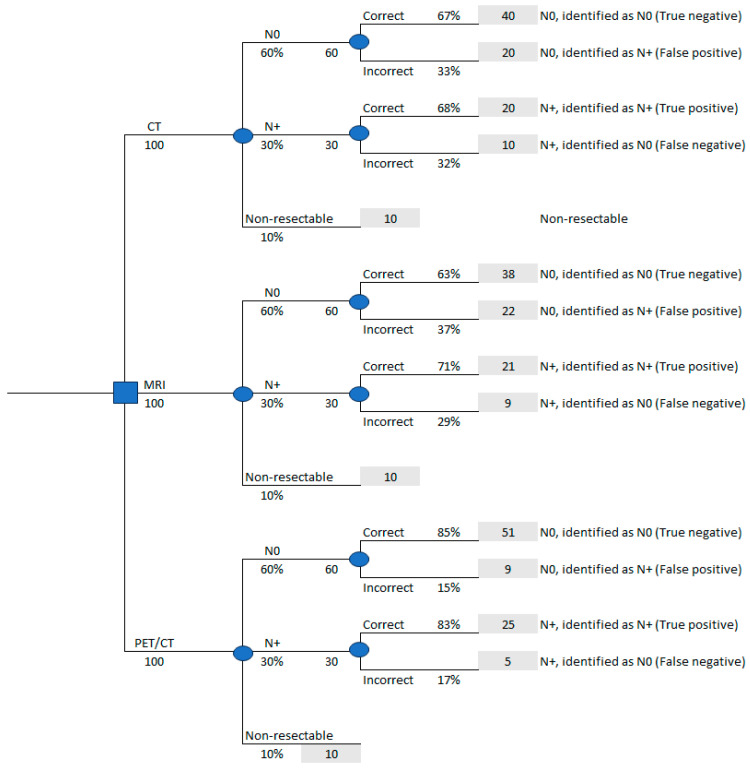
Decision analytic model including a decision tree. CT, MRI, and PET/CT diagnostic strategies are delineated, along with their outcomes. N+ and N0 denote the prevalence of lymph node disease (present or absent, respectively).

To simulate the natural history of HNC, a decision analytic model was constructed comprising two components. The first component of this decision analytic model was a decision tree, describing the initial decision-making process and the accuracy of testing, followed by a long-term Markov model. This model structure has been previously used in a similar economic evaluation [23] and was adapted herein to reflect the Greek healthcare setting. The decision tree represented the baseline staging of HNC and was based on three (3) different initial staging strategies, adapted from a previous relevant study [23] (Figure 1). These three initial staging strategies were as follows:

- Cervical and whole-body computed tomography (CT; including chest, upper and lower abdomen/pelvis), referred to hereafter as “CT.”
- Cervical MRI complemented with chest and upper–lower abdomen CT, collectively referred to as “MRI”.
- Whole-body fluoro-deoxy-glucose (FDG)-PET/CT, abbreviated as “PET/CT”.

**Figure 2:**
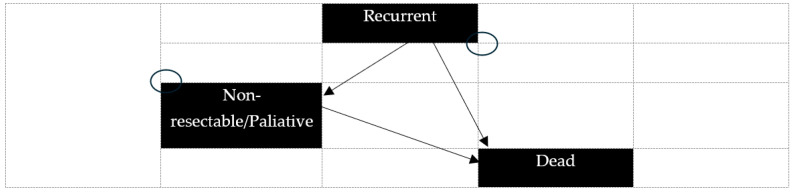
Markov model. Markov chain describing possible health states and patients; transitions after patients’ initial staging.

The Markov process included the possible transitions of patients among the health states of recurrence, non-resectable cancer/palliative care, and death. The Markov process started after the patients’ initial staging by each of the strategies under study (Figure 2). In this analysis, N0 refers to patients without cervical lymph node metastases, whereas N+ indicates patients with disease-involved cervical lymph nodes. The Markov cycle length was set to 1 year. Transitions between health states were assumed to occur midway through the cycle i.e., half-cycle correction was applied [24]. The “Death” state was an “absorbing state,” meaning it was the only state from which a patient could not transition to another, whereas other states were transient. Thus, patients were allocated to one of the following health states (Figure 1 and Figure 2):

(a) N0, correctly identified as N0 (True Negative);
(b) N0, incorrectly identified as N+ (False Positive);
(c) N+, correctly identified as N+ (True Positive);
(d) N+, incorrectly identified as N0 (False Negative);
(e) Presence of distant metastases (Metastatic);
(f) Disease recurrence (Recurrence);
(g) Death (Death).

The analysis focused on the initial staging of HNC patients, specifically those with a clinically N0 neck, meaning those with no evidence of metastatic involvement of cervical lymph nodes on initial clinical examination. These patients were expected to benefit most from an imaging modality with high diagnostic accuracy, such as PET/CT, regarding further surgical or other therapeutic decisions, including surgical planning and the decision to perform cervical lymphadenectomy and its extent (e.g., super-selective, selective, or radical) [25,26,27]. The prevalence of cervical lymph node involvement in such a cohort, ranges from 20 to 30% [26,27]. On the contrary, patients with locally advanced tumors have a significantly higher likelihood of cervical or distant metastases. The frequency of distant metastases in HNC patients varies between 2–18% across different series [9,27]. The analysis was based on the following assumptions:

- All True Negative patients underwent elective cervical lymphadenectomy and longitudinal follow-up post-surgery, incurring the corresponding costs.
- False Positive patients underwent unnecessary radical cervical lymphadenectomy followed by longitudinal follow-up.
- Patients with lymph node metastases (N+), whether True Positive or False Negative, underwent tumor resection along with cervical lymphadenectomy, supplemented with adjuvant chemotherapy, followed by longitudinal follow-up, as per the other groups.

For each group of True Negative, False Positive, True Positive, and False Negative patients, total survival (life years—LYs) and total costs at the end of the 10-year time horizon were calculated. These results were summed for each diagnostic strategy—CT, MRI, and PET/CT—after applying an annual discount rate of 3%.

The decision tree was populated using the diagnostic accuracy values reported by Linz et al. [28] (Table 1). Cost data for the CT, MRI, and PET/CT strategies were obtained from the National Organization of Primary Healthcare and Public Health (“EOPYY”), including the cost of the radiotracer (FDG) for PET/CT and costs of contrast agents for CT and MRI (Table 1). Data on age-dependent mortality unrelated to the disease for the Greek population of patients were obtained from the relevant survival tables provided by the European Health and Life Expectancy Information System (EHLEIS) [29]. The time horizon was set at 10 years consistent to the prototype analysis which this evaluation was based on [23]. A discount rate of 3% was used to convert costs and outcomes into present values [30] consistent with previous economic evaluations in Greece.

One-way deterministic and probabilistic sensitivity analysis was also conducted for the parameters of this model. The ranges of these analyses are presented in Table 1. For the probabilistic sensitivity analysis, beta distributions were used to reflect uncertainty around the mean value of diagnostic accuracy rates, whereas lognormal distributions were used for costs. The model was built in Microsoft Excel 365 (Microsoft^®^ Corporation (Redmond, WA, USA)).

## 3. Results

The results of the economic evaluation are summarized in Table 2 and Table 3. These present the total costs and LYs gained for the whole cohort of 100 patients with HNC for each alternative diagnostic strategy (CT, MRI, and PET/CT) and for every possible outcome of the initial decision tree shown in Figure 1.

Projecting the results to the level of a single patient over a 10-year time horizon, staging with PET/CT had a total cost of EUR 128,729, resulting in 6.171 LYs gained. The corresponding cost for MRI was EUR 128,779, yielding 6.170 LYs, while for CT, the cost was EUR 128,585, with 6.170 LYs gained. PET/CT was marginally more cost-effective and dominant over MRI, with a slightly lower cost and yielding marginally more LYs. The incremental cost-effectiveness ratio (ICER) of PET/CT compared to CT was calculated at EUR 144,984 for one (1) additional LY gained. The results of the comparisons among the different modalities and diagnostic strategies are summarized in Table 3.

**Figure 3:**
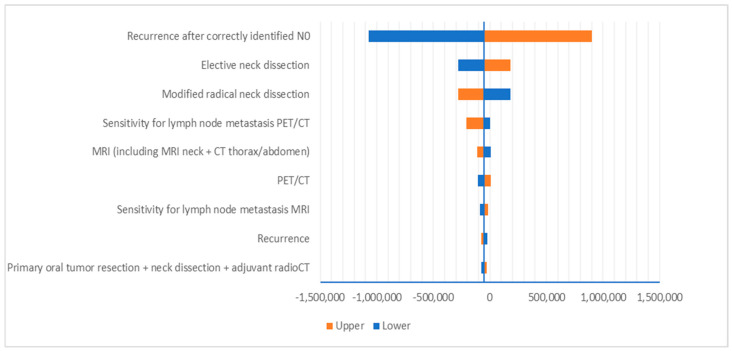
Deterministic sensitivity analysis results: PET/CT vs. MRI.

**Figure 4:**
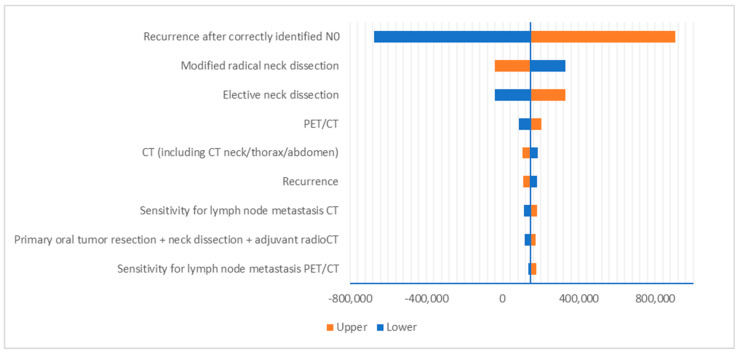
Deterministic sensitivity analysis results: PET/CT vs. CT.

**Figure 5:**
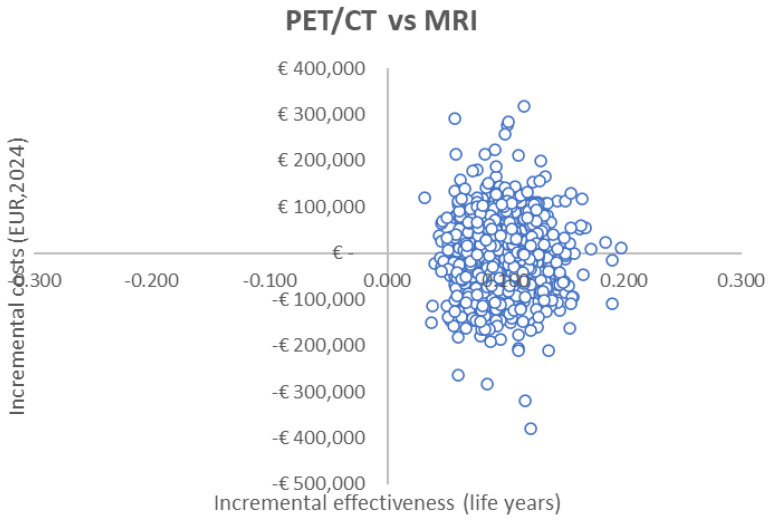

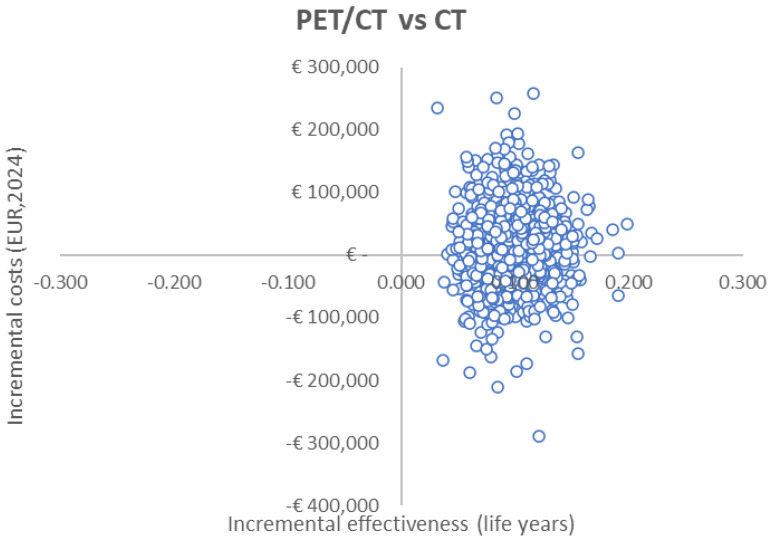
Probabilistic sensitivity analysis results for the ICERs of PET/CT vs. MRI or CT.

Deterministic sensitivity analysis showed that the ICERs are most sensitive to recurrence rates after patients were correctly identified as N0, which incurs substantial costs (Figure 3 and Figure 4). Probabilistic sensitivity analysis showed a 52% probability of dominance for PET/CT compared to MRI and 38% versus CT (Figure 5). The results for the ICER appeared to be sensitive due to the marginal differences in LYs gained among different modalities, i.e., the denominator of the ICER.

## 4. Discussion

This study demonstrated that PET/CT, MRI, and CT exhibit comparable clinical effectiveness with only minor differences in total cost per patient over a 10-year horizon (Table 3). Our findings provide context-specific insights complementing the existing literature. The quantitative estimates in our analysis differ from those reported by Burian et al. [23], using similar decision-making models, yet both series align in concluding small-scale differences between methods. Burian et al. reported that PET/CT dominated MRI and CT with lower costs and marginally more quality-adjusted life years (QALYs) gained, with 5.32 QALYs at USD 239,131 for PET/CT vs. 5.29 QALYs at USD 239,628 for CT vs. 5.30 QALYs at USD 240,001 for MRI. The differences in quantitative estimates between our study and that of Burian et al. underscore the impact of both healthcare setting-specific costs and clinical parameter values on final outcomes.

The epidemiological parameters chosen for the current analysis are more accurately aligned with the clinical profile of N0 patients. The lymph node disease incidence was set at 30%, and the metastatic disease incidence was set at 10%, reflecting more realistic scenarios for early-stage N0 patients compared with the higher rates (62% and 19%, respectively) in the Burian et al. series. The chosen sensitivity and specificity values were based on a prospective series of 135 oral cavity cancer patients with histological confirmation and similar incidence of nodal involvement [28], enhancing the clinical validity of our model and supporting its extrapolation to real-life clinical practice.

The present study is an initial effort to explore the cost-effectiveness of PET/CT within the Greek healthcare system and to compare it to other imaging modalities. The calculated ICER of EUR 144,984 per LY gained for PET/CT compared to CT exceeds commonly accepted European willingness-to-pay thresholds, even considering that our calculations refer to LYs rather than QALYs. Nonetheless, the ICER alone is insufficient as a single decision-making criterion. Cost-effectiveness thresholds vary considerably across countries, reflecting differences in models applied for the analyses, baseline assumptions, cost and clinical effectiveness data, societal demand for health, and prevailing budget constraints [34,35,36]. In Greece, there are no guidelines yet for the conduct of economic evaluations. Moreover, there are no published utilities that can be used in the calculation of QALYs. Moreover, although a drug HTA committee was established in 2018, there is currently no official cost-effectiveness threshold. Recently, Athanasakis et al. estimated the Greek ICER threshold at EUR 27,117 per QALY, using a supply-side econometric approach [34]. Specifically, they regressed quality-adjusted life expectancy against per capita public health expenditure over a 30-year period, while controlling relevant covariates. A systematic review of pharmacological interventions in Greece reported that most studies adopted ICER thresholds equal to one to three times the gross domestic product (GDP) per capita—approximately EUR 24,200 in 2025—with higher median thresholds (EUR 51,000) applied in oncological setting [37]. In the United Kingdom, the National Institute for Health and Care Excellence (NICE) applies thresholds between GBP 20,000–GBP 30,000 (about EUR 23,500–EUR 35,000) per QALY, to guide reimbursement and funding decisions for health technologies [38]. Evaluating the German health system’s cost-effectiveness threshold, the study by Gandjour applied a model-based approach using historical data on health expenditures and life expectancy [39]. He suggested a threshold of nearly EUR 90,000 per LY gained for innovative life-extending technologies without compromising the efficiency of the national healthcare system.

Given the established diagnostic superiority of PET/CT in HNC [9,28], it represents the method of choice for initial staging, facilitating resource optimization [23] and personalized treatment. PET/CT leads to individualized therapeutic decisions by identifying occult disease [16], guiding the extent of surgery or radiotherapy planning [9,27,40], and supporting adaptive, risk-stratified treatment approaches. PET/CT’s superior diagnostic accuracy may reduce false-positive cases and spare patients from unnecessary radical lymphadenectomies and concomitant surgical morbidity [41,42]. Moreover, its superior ability to detect distant metastases directly impacts treatment planning from locoregional to systemic cases.

While numerous studies have assessed the diagnostic accuracy of PET/CT in the initial staging, restaging, and treatment response evaluation of HNC, fewer studies have focused on its cost-effectiveness. Among these, a prospective randomized clinical trial in the United Kingdom demonstrated that PET/CT was a preferred strategy for evaluating patients with advanced HNC after combined chemoradiotherapy versus direct cervical lymphadenectomy. This approach resulted in fewer surgical interventions and immediate cost savings of GBP 1492 per patient, without compromising overall survival [41]. Extending these results over a long-term horizon, using a Markov model, showed that PET/CT remained cost-effective, saving GBP 1485 per patient with an additional benefit of 0.13 QALYs [43]. Another series concluded that the combination of PET/CT and MRI was preferable to invasive endoscopy for evaluating HNC patients after chemoradiotherapy, achieving similar diagnostic accuracy with fewer invasive procedures and a relatively low additional cost of EUR 927 per patient [44].

The results of our study may contribute to policymaking, since the Greek healthcare system currently operates under severe fiscal constraints and faces a complex, interconnected set of economic, structural, and global challenges. Since 2010, during the “memorandum era”, measures have been implemented to contain health costs, including the adoption of the electronic prescribing, the establishment of the “clawback and rebate” mechanisms to reduce pharmaceutical and diagnostic expenditures, and the unification of separate social health insurance funds into a single national entity (EOPYY) to optimize financial inflows and efficient collection of revenues [45,46]. Despite these ongoing efforts, limited funding is still putting pressure on the health system. Moreover, demographic aging is among the highest in Europe, and a concomitant increase in the prevalence of chronic and neoplastic diseases has been observed, exacerbated by suboptimal control of behavioral risk factors (e.g., smoking), the burden of climate change, and increasing hospital debts [47]. Given these challenges, efficient resource allocation through HTA and cost-effectiveness analyses are critical for sustainable health system management to improve efficiency and create fiscal space. Specifically in Greece, the use of PET/CT in HNC staging is reimbursed via EOPYY; thus, its optimal utilization contributes to efficient use of public resources and may result in rationalizing public spending without compromising public health outcomes.

Although they provide guidance, the results of our study should be interpreted with caution given the study’s inherent limitations. Since these results are derived from specific models and parameters, they are subject to variability, which was further explored with sensitivity analyses. Recurrence rates emerged as the most important parameter influencing the ICER. Recurrence increases costs, since it requires expensive multimodality treatment, and reduces patient survival, directly impacting the LYs gained. According to the parameters applied, there was significant cost differential between treating recurrent versus non-recurrent patients (EUR 32,269, Table 1). Deviations from the applied models and parameters will yield different quantitative outcomes and may lead to changed conclusions. Our model is characterized by simplicity in design and cannot entirely capture all potential events, outcomes, or complications associated with each therapeutic strategy at an individual level. It does not fully represent the complexity of routine clinical practice or individualized diagnostic (e.g., staging with both MRI and PET/CT) or treatment decisions.

Future research may employ advanced modeling approaches that accurately represent the complexity and variation in clinical trajectories of HNC. These models should incorporate prospective quality of life data as well as parameters relevant to reimbursement policies and health system structures from other countries, thereby enabling evaluation beyond the scope of the Greek national healthcare system. Future studies may evaluate different HNC subtypes according to anatomic disease sites (e.g., the nasopharynx, oropharynx, and larynx) and various biomarkers [48], such as human papilloma virus (HPV) status, TP53 mutations, and tumor mutational burden, to determine whether cost-effectiveness estimations vary across anatomically and molecularly defined patient subgroups. Additionally, quantitative PET parameters, including standardized uptake value (SUV) parameters, metabolic tumor volume (MTV), and radiomics, may be integrated into future analyses, as these indices correlate with treatment response, recurrence risk, and survival [49,50].

Another limitation of our study is the choice of overall survival (LYs) as the primary endpoint without incorporating quality-of-life data (QALYs). QALYs integrate both quantity and quality of life by multiplying LYs with utility weights ranging from 0 (death) to 1 (perfect health) [51,52]. In contrast, LYs quantify only survival benefit without accounting for functional status, treatment-related complications, or the burden of symptoms. LYs and QALYs cannot be used interchangeably, though it is acceptable to perform cost-effectiveness analyses without adjustment for quality of life [51]. HNC treatment substantially impairs quality of life by increasing morbidity and inducing a range of physical and functional complications, e.g., dysphagia, xerostomia, dysgeusia, speech impairment, pain, mucositis, infections, malaise, depression, etc. [53,54]. Utility weights for HNC survivors typically range from 0.70 to 0.85 depending on treatment modality, disease stage, and severity of complications [53]. PET/CT may affect quality of life by sparing patients from unnecessary treatment or lead other patients to more intensive and aggressive treatment. Prospective collection of utility data would be necessary for accurate conversion of LYs to QALYs, which would support more comprehensive economic evaluations [55].

## 5. Conclusions

Despite the limitations, this study represents the only work known to the authors examining the cost-effectiveness of PET/CT within the Greek national healthcare system using objective and quantitative criteria, specifically focusing on its application in the initial N-staging of HNC. Eventually, all different diagnostic strategies under investigation (PET/CT, MRI, and CT) demonstrated comparable clinical effectiveness in terms of LYs with only minor differences in total costs. Our results support the use of PET/CT in the initial staging of HNC, given the high diagnostic accuracy of the modality.

## Figures and Tables

**Table 1:** Model parameters.

Parameter	Mean	Lower	Upper	Distribution for PSA	Sources
Patient cohort size	100				
Initial patient age (years of age)	63				[23,31]
Pre-test probability of lymph node disease (prevalence)	30%	30.0%	27.0%	Beta	[26]
Pre-test probability of metastatic disease	10%	10.0%	9.0%	Beta	[9]
Annual discount rate	3%	2%	4%	Uniform	
Time horizon for Markov model (years)	10				
**Diagnostic accuracy values of different imaging modalities (N-staging)**					
Sensitivity of CT	67.6%	60.8%	74.4%	Beta	[28]
Specificity of CT	67.0%	57.4%	76.7%	Beta	
Sensitivity of MRI	70.6%	63.5%	77.7%	Beta	
Specificity of MRI	62.6%	52.7%	72.6%	Beta	
Sensitivity of PET/CT	83.3%	75.0%	91.6%	Beta	
Specificity of PET/CT	84.8%	76.3%	93.3%	Beta	
**Cost data (short-term, EUR 2024)**					
CT (neck + chest + upper–lower abdomen)	383	344	421	Lognormal	EOPYY tariffs
MRI (neck MRI + chest CT + upper–lower abdominal CT)	592	532	651	Lognormal	
PET/CT	580	522	638	Lognormal	
Elective nodal dissection	4271	3844	4698	Lognormal	DRG A01M
Modified radical nodal dissection	4271	3844	4698	Lognormal	
Tumor resection + nodal dissection + adjuvant chemotherapy	24,604	22,144	27,064	Lognormal	[32]
**Cost data (long-term, EUR 2024)**					
Follow-up post resection (×10 physician visits × EUR 40 per visit)	400	29,042	35,496	Lognormal	[32]
Metastatic disease *	32,269	29,042	35,496	Lognormal	
Recurrence *	32,269	29,042	35,496	Lognormal	[32,33]
**Transition probabilities for Markov model**					
Recurrence post correct diagnosis of Ν0 neck	12.8%	12.8%	11.5%	Beta	[23]
Recurrence after (erroneous) unnecessary radical neck dissection in patients with N0 neck	12.8%	12.8%	11.5%	Beta	
Recurrence following appropriate radical neck dissection in patients with N+ disease	12.8%	12.8%	11.5%	Beta	
Recurrence after incomplete (inappropriate) elective neck dissection in patients with N+ disease	15.7%	15.7%	14.1%	Beta	
Probability of death in Ν+ patients (initially and after recurrence)	17%	17.0%	15.3%	Beta	
Probability of death in metastatic disease	17%	12.8%	11.5%	Beta	

* Addition of palliative care costs to the cost of tumor resection + nodal dissection + adjuvant chemotherapy.

**Table 2:** Economic evaluation results for initial N-staging with CT, MRI, and PET/CT.

CT		No pts.	%	Costs	LYs
	N0, identified as N0 (true negative)	40.20	40%	EUR 5,896,285	322
	N0, identified as N+ (false positive)	19.80	20%	EUR 2,904,140	159
	N+, identified as N+ (true positive)	20.28	20%	EUR 1,962,457	69
	N+, identified as N0 (false negative)	9.72	10%	EUR 956,920	33
	Non-resectable	10.00	10%	EUR 1,100,456	34
	Total	100	100%	EUR 12,820,258	617
	Exam cost	EUR 38,264		EUR 12,858,522	617
**PET/CT**		**No pts.**	**%**	**Costs**	**LYs**
	N0, identified as N0 (true negative)	50.88	51%	EUR 7,462,761	408
	N0, identified as N+ (false positive)	9.12	9%	EUR 1,337,665	73
	N+, identified as N+ (true positive)	24.99	25%	EUR 2,418,235	85
	N+, identified as N0 (false negative)	5.01	5%	EUR 495,824	17
	Non-resectable	10.00	10%	EUR 1,100,456	34
	Total	100	100%	EUR 12,814,940	617
	Exam cost	EUR 58,000		EUR 12,872,940	617
**MRI**		**No pts.**	**%**	**Costs**	**LYs**
	N0, identified as N0 (true negative)	37.56	38%	EUR 5,509,066	301
	N0, identified as N+ (false positive)	22.44	22.4%	EUR 3,291,359	180
	N+, identified as N+ (true positive)	21.18	21%	EUR 2,049,548	72
	N+, identified as N0 (false negative)	8.82	9%	EUR 868,316	30
	Non-resectable	10.00	10%	EUR 1,100,456	34
	Total	100	100%	EUR 12,818,746	617
	Exam cost	EUR 59,156		EUR 12,877,902	617

**Table 3:** Results of the cost-effectiveness analysis and comparisons between CT, MRI, and PET/CT for the whole cohort of 100 patients.

	PET/CT	MRI	CT
**Costs (EUR, 2024)**	12,872,940.26	12,877,902.08	12,858,522.50
**Life years (LYs)**	617.11	617.01	617.01
**ΔCOST**		−4961.82	14,417.76
**ΔLY**		0.10	0.10
**ICER**		**PET/CT dominant**	144,984.89

ICER: incremental cost-effectiveness ratio.

## Data Availability

The Excel dataset is available upon reasonable request.

## Abbreviations

The following abbreviations are used in this manuscript:

CT	Computed tomography
DRG	Diagnosis-related group
EHLEIS	European Health and Life Expectancy Information System
EOPYY	National Organization for the Provision of Health Services (Greece)
FDG	Fluoro-deoxy-glucose (^18^F-fluorodeoxyglucose)
GDP	Gross domestic product
HNC	Head–neck cancer
HTA	Health technology assessment
HPV	Human papilloma virus
ICER	Incremental cost-effectiveness ratio
LY, LYs	Life year, life years
MRI	Magnetic resonance imaging
N0	No cervical lymph node metastases
N+	Presence of metastatic cervical lymph nodes
PET	Positron emission tomography
PET/CT	Positron emission tomography/computed tomography
QALY(s)	Quality-adjusted life year(s)
